# A method to estimate the number of neurons supporting visual orientation discrimination in primates

**DOI:** 10.12688/f1000research.12398.1

**Published:** 2017-09-26

**Authors:** Ruben Coen-Cagli, Ingmar Kanitscheider, Alexandre Pouget

**Affiliations:** 1 Department of Neuroscience, Albert Einstein College of Medicine, Bronx, New York City, NY, USA; 2 Department of Systems and Computational Biology, Albert Einstein College of Medicine, Bronx, New York City, NY, USA; 3 Center of Learning and Memory and Department of Neuroscience, The University of Texas at Austin, Austin, TX, USA; 4 Gatsby Computational Neuroscience Unit, University College London, London, UK; 5 Department of Brain and Cognitive Sciences, University of Rochester, Rochester, NY, USA; 6 Department of Basic Neuroscience, University of Geneva, Geneva, Switzerland

**Keywords:** visual cortex, vision, population coding

## Abstract

In this method article, we show how to estimate of the number of retinal ganglion cells (RGC), and the number of lateral genicular nucleus (LGN) and primary visual cortex (V1) neurons involved in visual orientation discrimination tasks. We reported the results of this calculation in Kanitscheider *et al*. (2015), where we were interested in comparing the number of neurons in the visual periphery versus visual cortex for a specific experiment. This calculation allows estimation of the information content at different stages of the visual pathway, which can be used to assess the efficiency of the computations performed. As these numbers are generally not readily available but may be useful to other researchers, we explain here in detail how we obtained them. The calculation is straightforward, and simply requires combining anatomical and physiological information about the macaque visual pathway. Similar information could be used to repeat the calculation for other species or modalities.

## Introduction

We would like to estimate:

1) the number of RGC and LGN neurons activated by a given visual stimulus; and

2) the number of V1 neurons that are activated by the same stimulus and are relevant to compute orientation.

We define as relevant those neurons that project to higher cortex, and separately consider the additional requirement that the neurons are tuned for orientation.

## Methods

### Upper bound on the number of cortical neurons

First, the number of neurons activated by a visual stimulus depends on the size and position of the image in the visual field. We use as a reference the setup of Dosher & Lu (1998) in experiments with human subjects, but the calculation reported below can be readily applied to visual stimuli of different size and position. Stimuli are presented parafoveally, at eccentricities between 2 and 5 degrees, and cover an area of 1×1 to 2×2 deg ^2^ in the visual field. To illustrate the calculation, we assume a 1×1 deg ^2^ stimulus at an eccentricity of 3 degrees.

The second factor is the volume of the cortex (surface x depth) that is activated by such stimuli. To estimate the surface we use cortical magnification factors (number of neurons per deg ^2^, as a function of eccentricity). We use here the results of Van Essen *et al*. (1984) who found that the following equation captured the relation between eccentricity ( *E*, in degrees) and cortical surface activated by a 1×1 deg ^2^ stimulus ( *M*, in mm ^2^/deg ^2^):

*M* = 103(0.82 + *E*) ^–2.28^              (1.1)

Hence, presenting a 1×1 deg ^2^ stimulus at an eccentricity of 3 degrees should activate a cortical surface of approximately 5 mm ^2^.

To determine cortical depth, we assume that the only V1 neurons used to solve a visual task are those that project to higher cortex, i.e. pyramidal neurons in layers 2/3/4B ( Kandel *et al.*, 2000, ch. 27). These layers constitute approximately 0.75 mm of cortical depth ( Peters & Rockland, 1994, ch. 1), and approximately 80% of neurons in V1 are excitatory ( Peters & Rockland, 1994, ch. 1), hence we consider an effective depth of 0.8×0.75=0.6 mm. Combining this with the estimated surface of 5 mm ^2^, we obtain an effective volume of 3 mm ^3^.

We then multiply this volume by cortical density, which in V1 is approximately 120,000 neurons/mm ^3^ ( O’Kusky & Colonnier, 1982). This leads to an estimate of 360,000 neurons/deg ^2^. This number represents an upper bound, assuming that all neurons contribute to decoding orientation regardless of their individual tuning for orientation. This is a reasonable assumption as long as the variability of untuned neurons is correlated with that of tuned neurons ( Zylberberg, 2017) or the tuning is not perfectly flat.

### Lower bound on the number of cortical neurons

To obtain also a lower bound, we consider the alternative that only neurons selective for orientation are relevant for orientation discrimination. Ringach *et al.* (2002) characterized the distribution of orientation selectivity in V1 across layers. Using bandwidth (half of the tuning curve width at $1/\sqrt{2}$ height) as a measure of selectivity, if we include only neurons with bandwidth smaller than 30 degrees we find that, across layers 2/3/4B, approximately 75% of the neurons satisfy the criterion. The threshold of 30 degrees is arbitrary; we chose a rather small threshold to obtain a lower bound. Combining the above estimates, the lower bound on the number of V1 neurons that can be used by downstream areas for orientation discrimination in the experimental setting considered here is 270,000. This is the estimate reported in Kanitscheider *et al.* (2015).

An additional consideration is that typical extracellular recordings with single electrodes, as in Ringach *et al.* (2002), tend to be biased towards neurons that are visually responsive (i.e. activity evoked by their preferred stimulus is substantially larger than spontaneous activity) and have high firing rates ( Olshausen & Field, 2005), raising the possibility that we overestimated the proportion of tuned neurons. Data recorded with chronically implanted multielectrode arrays ( Kelly *et al*., 2007), which do not suffer from those biases, indicate that the proportion of tuned neurons in L2/3 is consistent with Ringach *et al.* (2002). However, if some neurons were entirely silent throughout a recording sessions, i.e. they did not fire even a spontaneous action potential, they would go undetected, thus positively biasing the proportion of tuned neurons. We are not aware of direct estimates of the number of such neurons in macaque, but we can use as a guidance recent studies of rodent V1. Using calcium imaging, Ko *et al.* (2014) found that 55% of all neurons in a small volume are responsive to at least one visual stimulus, indicating that silent neurons represent at most 45% of the population. If we further assume that silent neurons do not contribute to orientation discrimination, we are left with a proportion of 0.75*0.55, or approximately 150,000 neurons. This represents a loose lower bound.

### Estimating the number of LGN and retinal neurons

The LGN volume and number of neurons activated by the same visual stimulus can be computed similarly from cell magnification factors derived by Malpeli *et al.* (1996) for parvocellular and magnocellular layers, respectively:

*N _P_* = 1,011,688(2.9144 + *E*) ^–2.6798^              

*N _M_* = 2,620.2(5.5638 + ( *E* – 1.8322) ^2^) ^–0.8012^              (1.2)

The above leads to an estimate of approximately 9,000 LGN neurons for a 1×1 deg ^2^ stimulus at an eccentricity of 3 degrees.

Lastly, to estimate the number of RGCs we used the cell magnification factors provided by Malpeli *et al.* (1996) figure 11 (based on Wässle *et al*., 1990), who assumed a constant fraction of RGCs projecting to LGN (90%) and binocular viewing conditions. We then interpolated linearly between the reported RGC densities at eccentricities of 5 degrees (3,000 cells/deg ^2^) and 2 degrees (8,500 cells/deg ^2^), and obtained an estimate of approximately 6,500 RGCs for a 1×1 deg ^2^ stimulus at an eccentricity of 3 degrees.

## Discussion

We have illustrated a method to estimate the number of neurons involved in a visual orientation discrimination task. The method involves considerations about experimental stimuli, including their size and position in the visual field, and considerations about the anatomy of the visual system, including magnification factors and neuron density. By considering different possible requirements for the subset of neurons that may be involved in the task, we have derived lower bounds on the estimates. Our results suggest an LGN-V1 expansion ratio between 17:1 and 40:1, similar to values reported previously for visual cortex ( DiCarlo *et al*., 2012; Olshausen & Field, 2004), and other sensory pathways ( Brecht & Sakmann, 2002; DeWeese *et al*., 2003; Mombaerts *et al*., 1996). Similarly, the RGC-V1 expansion ratio is between 23:1 and 55:1. The method could be readily applied to different stimuli and other visual areas.
