# Supplementary material for: A method to estimate the number of neurons supporting visual orientation discrimination in primates
Source: F1000Res. 2017 Sep 26;6:1752. [Version 1] doi: 10.12688/f1000research.12398.1 (PMC5750718; doi:10.12688/f1000research.12398.1)
Supplement: Peer review history

# Peer review: Referee response for version 1

**DOI:** 10.5256/f1000research.13427.r26423

**Published:** 2018-01-02

**Authors:** Denis G. Pelli

**Affiliations:** 1 Department of Psychology and Center for Neural Science, New York University, New York, NY, USA

The Coen-Cagli *et al*. paper is useful, showing a reasonable way to use known physiology and anatomy to estimate the number of neurons responding to a stimulus. This may be useful towards the long term goal of figuring out how the brain puts together the activity of multiple neurons to make perceptual decisions.

I have read this submission. I believe that I have an appropriate level of expertise to confirm that it is of an acceptable scientific standard.

# Peer review: Referee response for version 1

**DOI:** 10.5256/f1000research.13427.r28134

**Published:** 2017-11-28

**Authors:** Gregory W Schwartz

**Affiliations:** 1 Department of Ophthalmology, Feinberg School of Medicine, Northwestern University, Chicago, Illinois, USA

The authors provide a concise summary of the figures that go into estimating the number of neurons in retina, LGN, and V1 that are involved in a visual discrimination task. There are clearly a number of assumptions that go into such a calculation, but the authors do a good job of stating these assumptions explicitly and citing the relevant literature. One exception is the figure that 90% of RGCs project to LGN (specifically dorsal LGN in this case). This figure will also depend on eccentricity because the proportion of parasol and midget RGCs (which do project to dLGN) depends on eccentricity. In peripheral retina, the fraction of dLGN-projecting RGCs is far below 90% in primates. With this relatively simple fix, I think this short paper will provide a nice reference for calculations of cell counts in the early visual pathways.

I have read this submission. I believe that I have an appropriate level of expertise to confirm that it is of an acceptable scientific standard.
